# Maternal obesity phenotype, metabolic dysfunction, and preterm birth: a prospective birth cohort study

**DOI:** 10.3389/fnut.2025.1648996

**Published:** 2025-10-22

**Authors:** Jiayi Chen, Yecheng Miao, Qingxiu Li, Qian Zhang, Bin Sun, Zhengqin Wu, Wenjuan Liu, Junwei Liu, Huimin Shi, Haiyan Gao, Wei Li, Yibing Zhu, Haibo Li

**Affiliations:** ^1^Fujian Maternity and Child Health Hospital, College of Clinical Medicine for Obstetrics & Gynecology and Pediatrics, Fujian Medical University, Fuzhou, China; ^2^Department of Epidemiology and Health Statistics, School of Public Health, Fujian Medical University, Fuzhou, China; ^3^School of Clinical Medicine, Ningxia Medical University, Yinchuan, China; ^4^Division of Birth Cohort Study, Fujian Obstetrics and Gynecology Hospital, Fuzhou, China; ^5^Division of Birth Cohort Study, Fujian Children's Hospital, Fuzhou, China

**Keywords:** obesity metabolic phenotypes, metabolism, preterm birth, prospective cohort research, interaction analysis

## Abstract

**Introduction:**

The purpose of this research was to examine the relationship between metabolic obesity phenotypes and preterm birth (PTB) as well as the impact of obesity and metabolic abnormalities on PTB.

**Methods:**

A total of 20,259 pregnant singleton women participated in prospective birth cohort research conducted in China. Obesity metabolic phenotypes were categorized using pre-pregnancy body mass index (BMI) and metabolic state. Any delivery before 37 full weeks of gestation, as determined by the best obstetric estimate available, was considered PTB.

**Results:**

As the number of metabolically unfavorable components grows, so does the risk of developing PTB. Compared to women with a metabolically healthy normal weight, those who are normal weight and overweight (including obese) with metabolically unwell had an increased chance of having PTB (adjusted OR: 1.33 and 1.62, respectively). Additionally, additive interaction analysis revealed a significant interaction between overweight and metabolic unhealthiness for PTB risk (RERI = 0.41, AP = 0.24, SI = 2.22). People who are overweight and metabolically unwell have a 0.41 relative excess risk (which accounts for 24%) of PTB, and their combined risk is 2.22 times higher than that of those who are exposed to either risk alone.

**Conclusion:**

PTB risks are increased by metabolic abnormalities and overweight (including obese), and there are notable interaction effects between metabolic abnormalities and overweight (including obese) and PTB.

## 1 Introduction

Preterm birth (PTB), defined by the World Health Organization (WHO) as delivery before 37 completed weeks of gestation, represents a global clinical and public health challenge (1). It is associated with long-term adverse health effects in children and remains the leading cause of neonatal mortality and infant death (2). About 85% of these births are moderate (32–33 weeks) to late preterm babies (34–36 weeks), 10% are very preterm babies (28–31 weeks), and 5% are extremely preterm babies (<28 weeks) (3). Every year, about one million babies die from prematurity, and many survivors are disabled (4).

Numerous studies have demonstrated a link between obesity and preterm birth (5–7). Obesity can exacerbate the physiological inflammation associated with pregnancy (8). This inflammatory state, which is linked to both advanced maternal age and obesity, is a well-established risk factor for preterm birth (9). Additionally, common metabolic dysregulation, such as dyslipidemia, may be linked to inflammation and infection, and elevated inflammatory proteins may result in hypercholesterolemia, which may be linked to blood clot development and result in pregnancy issues such as placental abruption, which can exacerbate PTB (10, 11). Obesity is known as one of the most important public health concerns with a steadily increasing prevalence around the world, which is a well-known risk factor for many aspects of morbidity and mortality (12, 13), as well as causing a high economic burden to society (14). Current estimates suggest that by 2038 about 38% of the world's population is expected to be obese (15). In parallel with the increase in the general population, the prevalence of overweight and obesity also has increased in pregnant women (16). However, not all obese individuals show an equal health risk (17), some individuals may appear obesity but have normal metabolic conditions. They are defined as metabolically healthy obesity (MHO), a condition in which, despite the significant excess weight, traditional risk factors as insulin resistance, dyslipidemia, and hypertension are not present (18–20). Based on different metabolic conditions and body types, the population can be divided into metabolically unhealthy obesity (MUO), metabolically unhealthy normal weight (MUNW), and seven other types of obesity metabolic phenotypes.

Unfortunately, there are currently few thorough investigations on the relationship between obesity's metabolic abnormalities and pregnancy complications, with the majority of research on the metabolic phenotypes of obesity concentrating on cardiovascular disorders (21–23). In order to enrich this part of the research, we conducted a prospective birth cohort study among Chinese pregnant women. Our main goals are to quantify the separate and combined contributions of obesity and metabolic abnormalities to these pregnancy problems, as well as to clarify the relationship between metabolic obesity phenotypes and the incidence of PTB.

## 2 Methods

### 2.1 Study population

This prospective population-based cohort study was conducted to examine the relationship between metabolic obesity phenotypes and PTB. It was based on the Fujian Birth Cohort Study (FJBCS), which was initiated in November 2019 at the Fujian Maternal and Child Health Hospital in Fujian, China. As of June 2023, there were 25,538 patients with confirmed pregnancy outcomes. The final study requirements were met by 20,259 identified maternal mothers after excluding women who had diabetes or hypertension before conception or at baseline, had unclear pregnancy outcomes, had abortions, and had missed pre-pregnancy body mass index (BMI) data (Supplementary Figure S1). A comparison of the basic characteristics between participants and non-participants had shown in Supplementary Table S1. Due to the large sample size, the between-group differences were statistically significant, though the actual differences in baseline characteristics across populations remained within acceptable limits. The ethics committee of Fujian Provincial Maternal and Child Health Hospital authorized all study procedures (approval number: 2017KR-030), and each participant signed a written informed consent form. Additionally, we verified that every study was conducted in compliance with the Declaration of Helsinki.

### 2.2 Measurement of pre-pregnancy characteristics

In a face-to-face interview, participants completed a questionnaire about sociodemographic [maternal age, pre-pregnancy weight, ethnicity, assisted reproduction, gravidity, parity, marital status, income, work, inter-pregnancy interval, the season of delivery (spring, summer, autumn, and winter), and educational attainment] and lifestyle factors (history of smoking and alcohol consumption) when they were 10–12 weeks pregnant. The year of delivery (2019, 2020, 2021, and 2022) was obtained from the hospital information registry. Pre-pregnancy weight and height measurements were used to determine body mass index (BMI, kg/m^2^). An automated sphygmomanometer was used to measure the diastolic blood pressure (DBP) and systolic blood pressure (SBP). The enzymatic electrode method was used to measure the levels of fasting plasma glucose (FPG). Standardized enzymatic assays were used to examine serum lipid profiles, which included triglycerides (TG), total cholesterol (TC), low-density lipoprotein cholesterol (LDL-C), high-density lipoprotein cholesterol (HDL-C), apolipoprotein A1 (Apo-A1), and apolipoprotein B (Apo-B).

### 2.3 Definition of metabolic obesity phenotypes

The BMI of the study population is determined using the Working Group On Obesity in China established criteria (24), with “underweight” being defined as having a BMI of less than 18.5 kg/m^2^, “normal” as having a BMI between 18.5 and 24.0 kg/m^2^, “overweight” as having a BMI between 24.0 and less than 28.0 kg/m^2^, and “obese” as having a BMI greater than 28.0 kg/m^2^. We also performed sensitivity analyses using the WHO international BMI cut-offs. If a woman had any one of the following, she was said to have a metabolic factor: at the first prenatal booking, (i) the SBP was greater than 130 mmHg; (ii) the DBP was greater than 85 mmHg; (iii) the non-fasting capillary glucose was greater than 6.8 mmol/L; or (iv) dyslipidemia, which is defined as TC greater than 5.44 mmol/L, TG greater than 1.8 mmol/L, LDL-C greater than 3.96 mmol/L, HDL-C less than 1.04 mmol/L, or an apoB/apoA1 (apoB/apoA1) ratio greater than 0.8 (25). Metabolically healthy underweight (MHUW), metabolically unhealthy underweight (MUUW), metabolically healthy normal weight (MHNW), metabolically unhealthy normal weight (MUNW), metabolically healthy overweight (including obese; MHO), and metabolically unhealthy overweight (including obese; MUO) were the six groups into which all study participants were divided after combining their body weight phenotypes and metabolic conditions (2).

### 2.4 Definition of preterm birth

The PTB is defined as any birth before 37 completed weeks of gestation determined by the best available obstetric estimate, which mostly relied on an early pregnancy ultrasound in combination with the last menstrual period (LMP) (26): gestational age was determined using a combination of the last menstrual period (LMP) date and early ultrasound examination. For participants who underwent an ultrasound examination in early pregnancy ( ≤ 14 weeks), the measurement of crown-rump length was used as the primary method for estimating gestational age. For those without an early ultrasound scan, self-reported LMP date was used. After excluding iatrogenic preterm birth (such as that caused by placental abruption, placenta accreta, cervical cerclage, pulmonary hypertension, eclampsia, fetal distress, etc.), a sensitivity analysis was conducted with spontaneous preterm birth as the study outcome.

### 2.5 Statistical analyses

The median (interquartile range) was used to characterize skewed continuous data, whereas mean ± SD was used to express all regularly distributed continuous variables. Frequencies (%) were used to represent categorical variables. To check for differences between the various groups of obesity metabolic phenotypes, we employed the Kruskal–Wallis *H* test (skewed distribution), One-Way ANOVA test (normal distribution), or Chi-square (categorical variables) test.

Using multivariable logistic regression analysis, the adjusted odds ratio (aOR) and 95% confidence interval (CI) for the beginning of PTB were evaluated. Known to affect metabolic status or be linked to PTB, covariates were chosen beforehand and modified in the logistic regression models. Maternal age, ethnicity, gravidity, parity, assisted reproduction, alcohol and tobacco use, marital status, and educational attainment were among these factors. Missing covariates were addressed using imputation by chained equations.

Pre-specified stratified analyses were based on subgroup characteristics (parity, pregnancy type, and maternal age). To check for multiplicative interaction between subgroups, the likelihood ratio test was employed. Furthermore, additive interaction was evaluated using two indices: the relative excess risk due to interaction (RERI), and the attributable proportion (AP) owing to interaction and verified RERI, AP, and the SI by using the delta method. If the 95% CIs for RERI and AP do not overlap 0, the interaction between them is considered statistically significant. Additionally, the estimated OR for PTB of a logistic model with a metabolic abnormalities-BMI interaction term was utilized to create an interaction spline with four knots using the R package “interaction RCS.” The R Statistical Software (Version 4.2.2, http://www.R-project.org, The R Foundation) were used for all analyses. Statistical significance was defined as a two-sided *P* value <0.05.

## 3 Results

### 3.1 Baseline characteristics

Table 1 lists the baseline demographic and clinical characteristics stratified by obesity metabolic phenotypes among 20,259 women during their first pregnancy. The participation rate among eligible women was 75%. Those who defined as MUO were more likely to be older and to drink alcohol than those who had a typical pregnancy. Compared to women with other metabolic phenotypes of obesity, individuals with MUO had lower levels of HDL (1.7 ± 0.4 mmol/L) but greater pre-pregnancy BMI (26.8 ± 6.7 kg/m^2^), blood pressure (SBP/DBP: 122.0 ± 10.9/73.9 ± 9.2 mmHg), FPG (5.2 ± 1.3 mmol/L), TC (6.5 ± 1.3 mmol/L), and TG [3.8 (3.0–5.0) mmol/L].

**Table 1:** Demographic, metabolic and clinical variables in pregnant women by obesity metabolic phenotype group.

**Maternal characteristics**	**Total (20,259)**	**Obesity metabolic phenotype**				***P* value**
		**MHUW/MHNW (12,030)**	**MUUW/MUNW (5,196)**	**MHO (1,459)**	**MUO (1,574)**	
Maternal age, years	30.3 ± 3.9	29.9 ± 3.8	30.7 ± 4.0	30.9 ± 4.0	31.4 ± 4.2	<0.001
Ethnicity-Han, *n* (%)	19,823 (97.8)	11,774 (97.9)	5,084 (97.8)	1,428 (97.9)	1,537 (97.6)	0.634
Educational-University level, *n*(%)	14,537 (71.9)	8,812 (73.3)	1,001 (68.7)	3,693 (71.2)	1,031 (65.6)	<0.001
Marriage, *n* (%)	19,231 (94.9)	11,368 (94.5)	4,941 (95.1)	1,405 (96.3)	1,517 (96.4)	0.001
Smoking, *n* (%)	410 (2.0)	229 (1.9)	93 (1.8)	45 (3.1)	43 (2.7)	0.005
Alcohol status, *n* (%)	16,235 (80.1)	9,610 (79.9)	4,209 (81)	1,164 (79.8)	1,252 (79.5)	<0.001
**Income**						<0.001
<0.9 w (monthly)	8,364 (41.3)	4,989 (39.7)	408 (43.6)	2,407 (42.6)	560 (50.1)	
≥0.9 w (monthly)	11,895 (58.7)	7,565 (60.3)	527 (56.4)	3,246 (57.4)	557 (49.9)	
Work (employees or technical staff)	14,759 (72.9)	9,326 (74.3)	679 (72.6)	4,032 (71.3)	722 (64.6)	<0.001
**Pregnancy interval (year)**						<0.001
<1	8,870 (43.8)	5,850 (46.6)	369 (39.5)	2,303 (40.7)	348 (31.2)	
1–2	2,630 (13.0)	1,564 (12.5)	125 (13.4)	740 (13.1)	201 (18)	
≥2	8,759 (43.2)	5,140 (40.9)	441 (47.2)	2,610 (46.2)	568 (50.9)	
Assisted reproduction, *n* (%)	1,424 (7.0)	684 (5.7)	489 (9.4)	95 (6.5)	156 (9.9)	<0.001
**Gravidity**, ***n*** **(%)**						<0.001
1	8,870 (43.8)	5,652 (47)	2,159 (41.6)	567 (38.9)	492 (31.3)	
2	6,143 (30.3)	3,608 (30)	1,538 (29.6)	451 (30.9)	546 (34.7)	
≥3	5,246 (25.9)	2,770 (23)	1,499 (28.8)	441 (30.2)	536 (34.1)	
**Parity**, ***n*** **(%)**						<0.001
0	12,124 (59.8)	7,565 (62.9)	2,976 (57.3)	807 (55.3)	776 (49.3)	
1	7,293 (36.0)	4,026 (33.5)	1,986 (38.2)	573 (39.3)	708 (45)	
≥2	842 (4.2)	439 (3.6)	234 (4.5)	79 (5.4)	90 (5.7)	
Pre-pregnancy BMI, kg/m^2^	21.2 ± 4.1	20.1 ± 2.0	20.7 ± 2.0	26.5 ± 8.0	26.8 ± 6.7	<0.001
SBP, mmHg	114.5 ± 11.1	111.8 ± 9.8	118.6 ± 12.1	114.6 ± 9.8	122.0 ± 10.9	<0.001
DBP, mmHg	69.3 ± 9.9	67.5 ± 7.9	72.2 ± 13.1	69.5 ± 7.9	73.9 ± 9.2	<0.001
FPG, mmol/L	5.0 ± 1.2	5.0 ± 1.1	5.1 ± 1.2	5.1 ± 1.3	5.2 ± 1.3	<0.001
TC, mmol/L	6.6 ± 1.2	6.5 ± 1.1	7.0 ± 1.4	6.1 ± 1.1	6.5 ± 1.3	<0.001
TG, mmol/L	3.2 (2.5, 4.1)	3.0 (2.4, 3.8)	3.6 (2.8, 4.7)	3.1 (2.5, 3.8)	3.8 (3.0, 5.0)	<0.001
HDL, mmol/L	1.8 ± 0.4	1.8 ± 0.4	1.8 ± 0.4	1.8 ± 0.3	1.7 ± 0.4	<0.001
LDL, mmol/L	3.6 ± 1.0	3.6 ± 0.9	3.8 ± 1.2	3.3 ± 0.9	3.5 ± 1.1	<0.001
Apo A1, g/L	1.5 ± 0.3	1.5 ± 0.3	1.6 ± 0.3	1.4 ± 0.3	1.5 ± 0.3	<0.001
Apo B, g/L	0.7 ± 0.2	0.7 ± 0.1	0.8 ± 0.2	0.7 ± 0.1	0.9 ± 0.2	<0.001
Apo B/Apo A1	0.5 ± 0.1	0.5 ± 0.1	0.5 ± 0.1	0.5 ± 0.1	0.6 ± 0.2	<0.001
Hypertensive, *n* (%)	2,057 (10.2)	0 (0)	1,539 (29.6)	0 (0)	518 (32.9)	<0.001
Hyperglycemia, *n* (%)	360 (1.8)	0 (0)	229 (4.5)	0 (0)	131 (8.4)	<0.001
Hyperlipidemia, *n* (%)	5,231 (25.8)	0 (0)	3,965 (76.3)	0 (0)	1,266 (80.4)	<0.001
Metabolic unhealthy, *n* (%)	13,489 (66.6)	12,030 (100)	0 (0)	1,459 (100)	0 (0)	<0.001
Preterm birth, *n* (%)	1,000 (4.9)	516 (4.3)	287 (5.5)	72 (4.9)	125 (7.9)	<0.001

BMI, body mass index; SBP, systolic blood pressure; DBP, diastolic blood pressure; FPG, fasting plasma glucose; TC, total cholesterol; TG, triglyceride; HDL, high-density lipoprotein; LDL, low-density lipoprotein; Apo A1, apolipoprotein A1; Apo B, apolipoprotein B; MHUW, metabolically healthy underweight; MUUW, metabolically unhealthy underweight; MHNW, metabolically healthy normal weight; MUNW, metabolically unhealthy normal weight; MHO, metabolically healthy overweight (including obese); MUO, metabolically unhealthy overweight (including obese). Continuous variables with normal distributions are expressed as the mean-standard deviation, whereas those with non-normal distributions are presented as the median and interquartile range.

### 3.2 The odds ratios (ORs) for preterm birth based on the body mass index, metabolic status, and metabolic components

Analysis revealed a significant association between metabolic health status and the risk of preterm birth: among 20,259 participants, compared to metabolically healthy women, the risk of preterm birth increases by 28% in women with one metabolically unhealthy component (aOR = 1.28, 95% CI: 1.11–1.47, *P* = 0.001), and the risk increases by 86% in women with two metabolically unhealthy components (*P* < 0.001). It is worth noting that the impact of hyperlipidemia on the risk of preterm birth is particularly significant, with an aOR of 1.45 (95% CI: 1.26–1.66, *P* < 0.001) and the risk of preterm birth in metabolically unhealthy women 34% higher than in metabolically healthy women (*P* < 0.001). Interestingly, the analysis showed that the effects of having three metabolically unhealthy number components on the occurrence of PTB were not statistically significant (Table 2). Similar results were also observed in the analysis of spontaneous preterm birth (Supplementary Table S2).

**Table 2:** The odds ratios (ORs) for PTB according to the body mass index, metabolic components, and metabolic status.

**Variable**	**Total, *n***	**PTB, *n* (%)**	**Crude**		**Adjusted**	
			**OR (95%CI)**	***P*** **value**	**OR (95%CI)**	***P*** **value**
**Body mass index**						
Underweight	3,114	135 (4.3)	0.91 (0.75–1.1)	0.34	0.99 (0.82–1.19)	0.892
Normal weight	14,112	668 (4.7)	1 (Ref)		1 (Ref)	
Overweight (including obese)	3,033	197 (6.5)	1.40 (1.19–1.65)	<0.001	1.33 (1.12–1.57)	0.001
**Metabolic components**						
**Hypertensive**						
No	18,202	877 (4.8)	1 (Ref)		1 (Ref)	
Yes	2,057	123 (6)	1.26 (1.03–1.53)	0.021	1.23 (1.01–1.5)	0.036
**Hyperlipidemia**						
No	15,028	657 (4.4)	1 (Ref)		1 (Ref)	
Yes	5,231	343 (6.6)	1.53 (1.34–1.76)	<0.001	1.45 (1.26–1.66)	<0.001
**Hyperglycemia**						
No	19,574	950 (4.9)	1 (Ref)		1 (Ref)	
Yes	360	22 (6.1)	1.28 (0.82–1.97)	0.273	1.14 (0.74–1.77)	0.548
**Number of metabolically unhealthy components**						
0	13,489	588 (4.4)	1 (Ref)		1 (Ref)	
1	5,930	340 (5.7)	1.33 (1.16–1.53)	<0.001	1.28 (1.11–1.47)	<0.001
2	802	68 (8.5)	2.03 (1.56–2.64)	<0.001	1.86 (1.43–2.42)	<0.001
3	38	4 (10.5)	2.58 (0.91–7.3)	0.074	2.22 (0.78–6.29)	0.134
**Metabolic status**						
Metabolically healthy	13,489	588 (4.4)	1 (Ref)		1 (Ref)	
Metabolically unhealthy	6,770	412 (6.1)	1.42 (1.25–1.62)	<0.001	1.34 (1.18–1.53)	<0.001

Models were adjusted for age, ethnicity, education, marriage, smoking, alcohol status, gravidity, parity, assisted reproduction, income, work and inter-pregnancy. PTB, preterm birth; OR, odds ratio; aOR, adjusted odds ratio.

### 3.3 The association of obesity metabolic phenotypes with preterm birth

**Figure 1:**
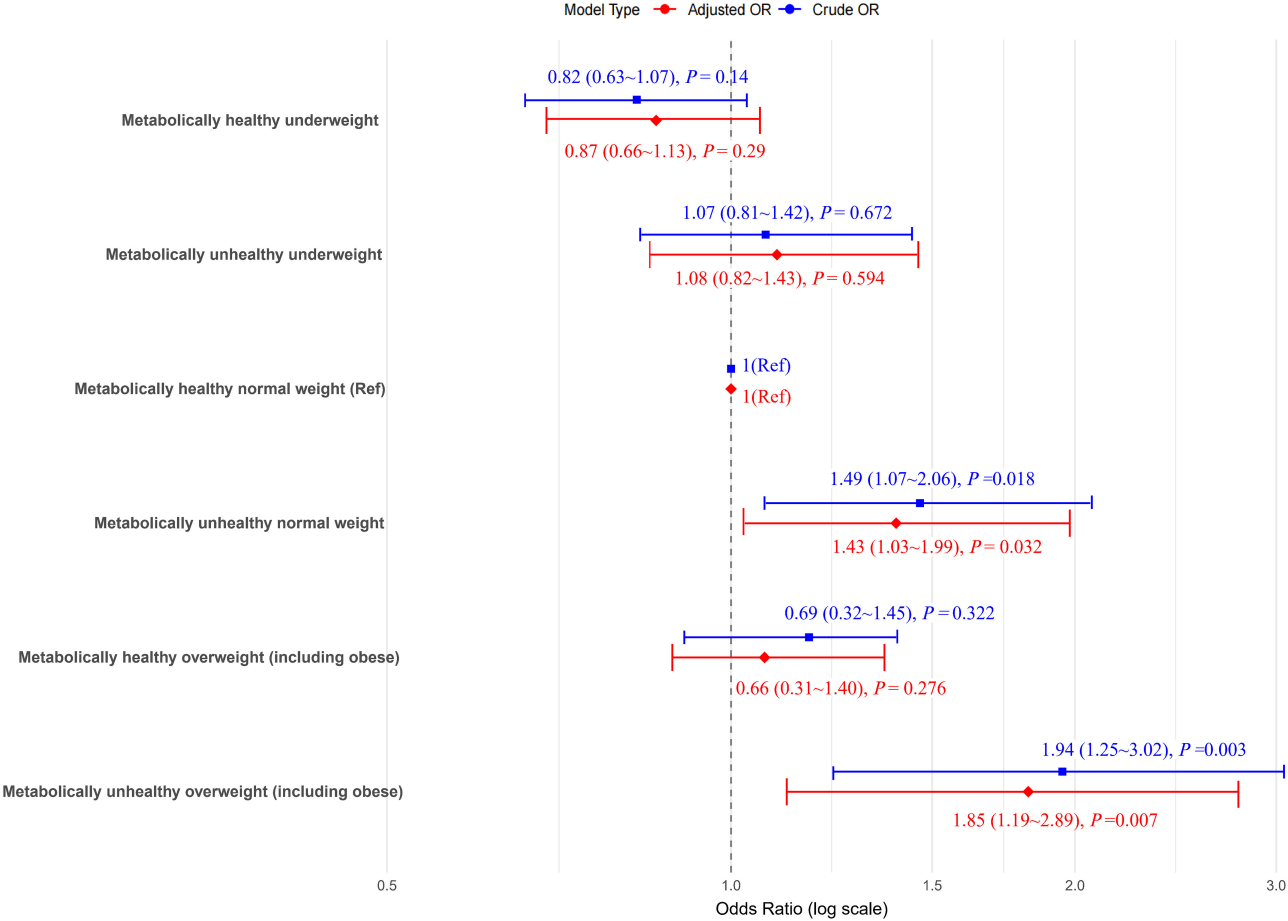
The odds ratios (ORs) for preterm birth (PTB) according to the metabolic body weight phenotypes. Models adjusted for age, ethnicity, education, marriage, smoking, alcohol status, gravidity, parity, assisted reproduction, income, work and inter-pregnancy. The blue icons in the model type are boxes and the red ones are diamonds.

Compared to MHNW women, MUO women showed the highest risk of developing PTB after full adjustment, with aOR of 1.85 (95% CI: 1.19–2.89). The risks of developing PTB were significantly increased by metabolically unhealthy which showed that women with MUNW had 43% higher PTB chances, with aORs of 1.43 (95% CI: 1.03–1.99) than MHNW. However, compared to MHNW women, the preterm birth risk for MHUW, MHO, and MUUW women was not observed to be statistically significant (Figure 1).

### 3.4 Association of metabolic phenotypes with preterm birth across body weight categories

In women with normal weight, the risk of preterm birth is higher in metabolically unhealthy women compared to metabolically healthy individuals, with an aOR of 1.33 (95% CI: 1.13–1.57). Similarly, in overweight (including obese) women, the incidence of preterm birth is higher in metabolically unhealthy women compared to metabolically healthy women, with an aOR of 1.62 (*P* = 0.002; Table 3). Analysis of spontaneous preterm birth revealed similar results (Supplementary Table S3).

**Table 3:** Relationship of metabolic phenotypes and PTB in different body weight phenotypes.

**Variable**	**Total, *n***	**PTB, *n* (%)**	**Crude**		**Adjusted**	
			**OR (95%CI)**	***P*** **value**	**aOR (95%CI)**	***P*** **value**
Metabolically healthy underweight	2,446	110 (4.5)	1 (Ref)		1 (Ref)	
Metabolically unhealthy underweight	668	25 (3.7)	0.83 (0.53–1.29)	0.397	0.81 (0.52–1.26)	0.35
Metabolically healthy normal weight	9,584	406 (4.2)	1 (Ref)		1 (Ref)	
Metabolically unhealthy normal weight	4,528	262 (5.8)	1.39 (1.18–1.63)	<0.001	1.33 (1.13–1.57)	<0.001
Metabolically healthy overweight (including obese)	1,459	72 (4.9)	1 (Ref)		1 (Ref)	
Metabolically unhealthy overweight (including obese)	1,574	125 (7.9)	1.66 (1.23–2.24)	0.001	1.62 (1.19–2.19)	0.002

Models were adjusted for age, ethnicity, education, marriage, smoking, alcohol status, gravidity, parity, assisted reproduction, income, work and inter-pregnancy. OR, odds ratio; aOR, adjusted odds ratio.

### 3.5 Interaction analysis of the effects of body weight status and metabolic phenotypes on preterm birth

**Figure 2:**
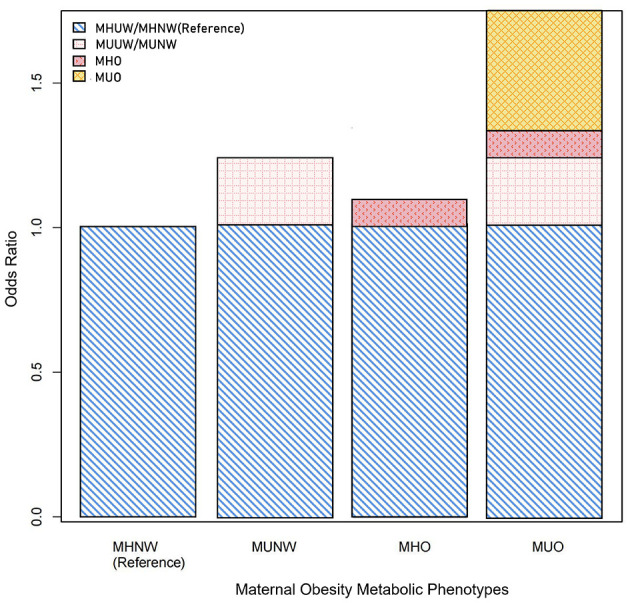
Additive interactions between overweight (including obese) and metabolically unhealthy on the risk of preterm birth (PTB). Models were adjusted for age, ethnicity, education, marriage, smoking, alcohol status, gravidity, parity, assisted reproduction, income, work and inter-pregnancy.

**Figure 3:**
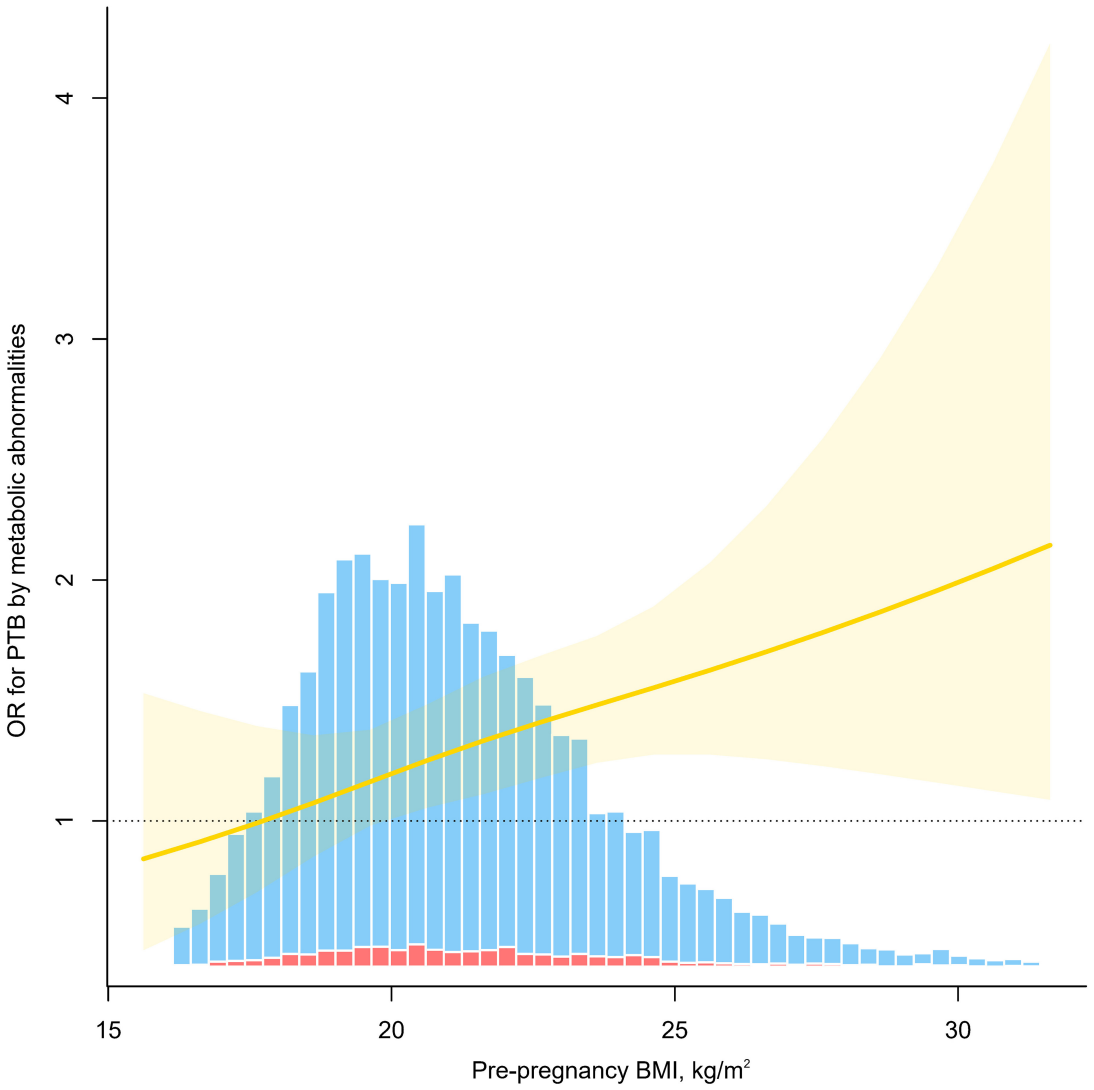
Odds ratio (OR) for preterm birth (PTB) by metabolic abnormalities as a function of pre-pregnancy BMI. The solid red line indicates the estimated OR, the yellow shading indicates the 95% confidence interval, and the bar diagram indicates the distribution of the population, where the red columns are for those who developed PTB and the blue columns are for those who did not. Models were adjusted for age, ethnicity, education, marriage, smoking, alcohol status, gravidity, parity, assisted reproduction, income, work and inter-pregnancy.

Significant additive interactions and multiplicative interactions were found between body weight status and metabolic phenotypes on the risk of PTB (Table 4, Figure 2). Three measures of additive interaction between being overweight (including obese) and metabolically unhealthy (RERI, AP) showed a relative excess risk of 0.41, with an AP of 0.24 (95% CI: 0.01–0.46), indicating that the combined effect of these two risk factors may result in a 24% increased risk of PTB (Table 4, Figure 2). According to the interaction spline, metabolic problems were linked to PTB across all BMI ranges, however the relationship was noticeably stronger at higher BMI values (Figure 3).

**Table 4:** Interaction analysis of the effects of overweight (including obese) and metabolically unhealthy on PTB.

**Measures**	**OR/Estimates**	**95% CI**	***P* value**
**Obesity metabolic phenotype**			
MHNW	1 (Ref)		
MUNW	1.24	1.07–1.44	<0.001
MHO	1.09	0.85–1.41	0.51
MUO	1.76	1.43–2.16	<0.001
**Subgroup analysis**			
**Metabolically unhealthy on PTB**			
Normal weight	1.24	1.07–1.44	<0.001
Overweight	1.60	1.18–2.16	<0.001
**Overweight on PTB**			
Metabolically healthy	1.09	0.85–1.41	0.49
Metabolically unhealthy	1.41	1.13–1.75	<0.001
**Interaction analysis**			
Multiplicative interaction	1.29	0.92–1.80	0.14
**Additive interaction**			
RERI	0.41	0.00–0.85	0.03
AP	0.24	0.01–0.46	0.02
SI	2.22	0.76–6.48	<0.001

Multiplicative interaction of the effects of overweight (including obese) and metabolically unhealthy on preterm birth was assessed by including the main effects of them and the product term in the model and the P_interaction_ was represented by the P-value of product term. RERI, AP were used to indicate additive interaction of overweight (including obese) and metabolically unhealthy on preterm birth. Models were adjusted for age, ethnicity, education, marriage, smoking, alcohol status, gravidity, parity, assisted reproduction, income, work and inter-pregnancy. MHNW, metabolically healthy normal weight; MUNW, normal weight with metabolic abnormalities; MHO, overweight (including obese) without metabolic abnormalities; MUO, overweight (including obese) with metabolic abnormalities.

### 3.6 Subgroup analysis and sensitivity analyses

**Figure 4:**
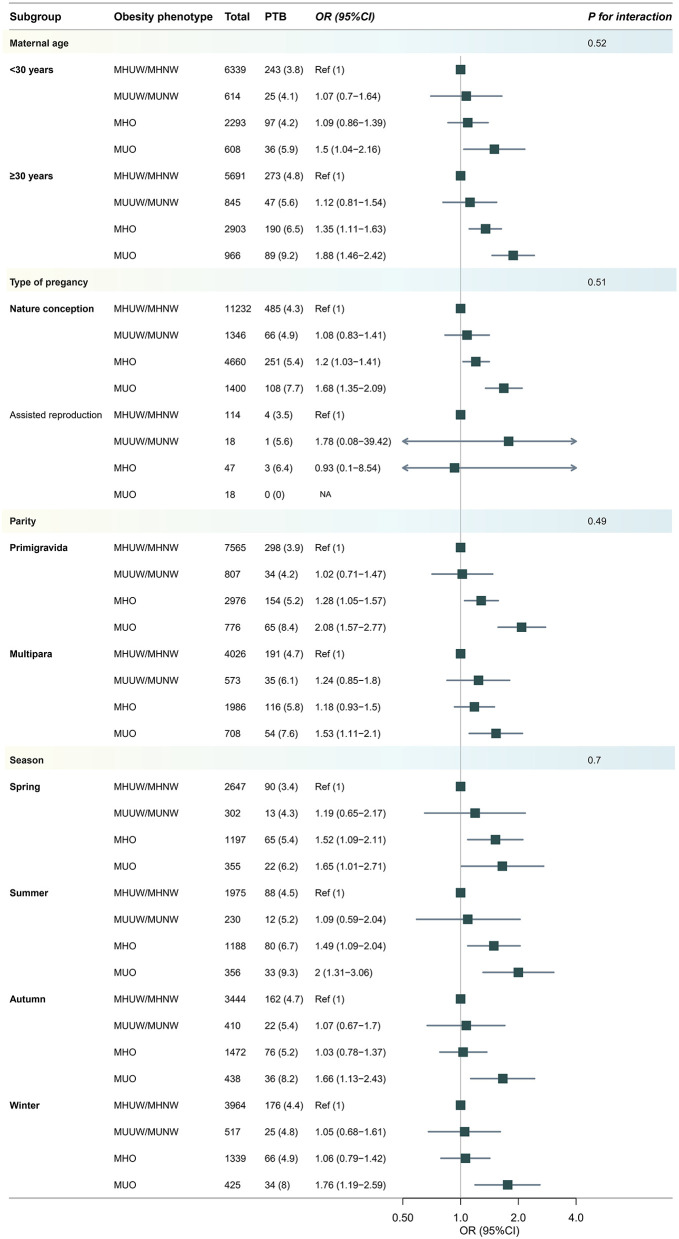
Subgroup analysis for risk of developing preterm birth (PTB) according to body weight phenotype. Models were adjusted for age, ethnicity, education, marriage, smoking, alcohol status, gravidity, parity, assisted reproduction, income, work and inter-pregnancy.

Additional subgroup analyses concerning the role of maternal age, parity and assisted reproduction, the year of delivery and the season of delivery are presented and shown in the Figure 4. Interestingly, the interaction tests showed that these stratification variables had no modification effects (all interaction *P*-values >0.05), indicating that the observed associations between metabolic obesity indicators and PTB were unaffected by the mother's age, reproductive history, or method of conception. We also performed supplementary sensitivity analyses using the WHO international BMI cut-offs to define obesity (Supplementary Tables S4–S7), and revealed pattern consistent results.

## 4 Discussion

The present study showed that women who were overweight, including obese, were more likely to develop PTB than women who were normal weight, and that people with metabolic abnormalities were more likely to develop PTB even if they had the same weight phenotype. We also found that there was an additive and multiplicative interaction between PTB and metabolic abnormalities and overweight.

Pregnant women who are obese are at greater risk of a variety of pregnancy-related complications compared with women of normal BMI (27). Several studies have shown that the risk of PTB is positively related to obesity. A large prospective cohort study from China showed that compared to women with normal weight, women with overweight or obesity before pregnancy had an increased risk of preterm birth. In addition, the greatest risk of extremely preterm birth was observed in obese women (7). Ju et al. (28) found that overall maternal obesity (BMI ≥ 30.0 kg/m^2^) and extreme obesity (BMI ≥ 40.0 kg/m^2^), were both associated with higher rates of prematurity after controlling for other confounders including maternal race, age, socioeconomic status, and smoking during pregnancy. Obesity can enhance the inflammatory status induced by pregnancy (29), which is characterized by a state of chronic inflammation, oxidative stress, and dysregulated adipokine secretion and will promotes the release of damage-associated molecular patterns (DAMPs). These DAMPs amplify uterine and chorioamnionic inflammation via inflammasome activation, potentially leading to intra-amniotic inflammation, which increase the risk of spontaneous extremely preterm birth. In addition, Obesity compromises placental function through multiple interconnected pathways: chronic inflammation and oxidative stress directly damage placental cells and impair nutrient exchange; epigenetic alterations (e.g., DNA methylation) disrupt gene expression patterns critical for placental development; and dysregulation of metabolic mediators (e.g., leptin, apelin) disrupts vascular tone and angiogenesis, while maternal vascular malperfusion is a well-established risk factor for indicated preterm birth (30). Collectively, these mechanisms may lead to PTB (31).

Numerous diseases are directly linked to metabolic issues, which raises the risk of developing a variety of ailments. Despite having a normal BMI, poor metabolic health still raises the risk of many diseases. Numerous studies have linked metabolic abnormalities to both poor pregnancy outcomes and chronic (32–34) and cardiovascular disease (35, 36). Numerous studies have demonstrated that women with a history of hypertension illnesses during pregnancy are more likely to have cardiovascular disease later in life than those with normotensive pregnancies (37). A study including more than 600,000 women in Norway showed that hypertension during pregnancy was associated with an increased risk of subsequent CVD in comparison with normotensive pregnancy and the highest risk was observed when hypertension was combined with small-for-gestational-age infants and/or preterm delivery (38). Inflammation and infection may help explain the association between metabolic abnormalities and PTB. For example, the lipid changes could relate to infammation and infection, and that hypertriglyceridemia may be considered part of the innate immunity and that increased inflammatory proteins may cause hypercholesterolemia. Studies have shown that high cholesterol may contribute to the formation of blood clots, which can lead to complications during pregnancy, such as placental abruption, which can promote PTB (11). It is noteworthy that our results imply that the development of PTB is significantly influenced by the metabolic state prior to pregnancy. PTB is more common in metabolically unhealthy women than in those who are metabolically healthy, and the risk of PTB rises as the number of metabolically unfavorable components rises. This demonstrates the significance of metabolic status as a risk factor for obstetric issues and validates the results of other previous studies (1, 39–41). Meanwhile, among individuals with metabolic abnormalities, the risk of PTB is significantly increased for both normal-weight and overweight women, which illustrates the importance of the metabolic screening in normal-weight women and to reduce the risk of metabolic abnormalities by improving lifestyle habits, such as maintaining a balanced diet and engaging in regular physical activity. For high-risk individuals with metabolic abnormalities, medications like metformin can be used to lower the risk of developing metabolic diseases.

In this sizable population cohort study, we also evaluated the combined impact of obesity and metabolic disorders on PTB. Compared with MHNW women, we discovered that MUNW and MUO women were more likely to have PTB, which was same as the previous study (25). Among overweight women, PTB was much more common in those with metabolic problems. It is worth noting that among individuals with metabolic abnormalities, the risk of PTB is significantly increased for both normal-weight and overweight women. Interaction analysis shows that overweight and metabolic abnormalities have both additive and multiplicative interactions on the occurrence of PTB which demonstrated that the additive interaction accounts for 24% of PTB events in those exposed to both risk factors (metabolically unhealthy and overweight).

The large sample size and the evaluation of interactions between body weight status and metabolic phenotypes on PTB, as well as a quantitative assessment of the association between obesity and metabolic abnormalities, were the primary strengths of our work. However, the present study has several limitations. First, the pre-pregnancy weight used to calculate BMI is provided by the study population, which may affects the accuracy of the BMI calculation. In addition, the BMI cannot show the distribution of body fat or differentiate between lean and fat mass (42). Second, several subgroup analyses were performed with a limited number of studies, making it difficult to achieve a firm conclusion about the findings. Another potential limitation of this study is its hospital-based design. As the sample was recruited from clinical settings rather than the general community, it is susceptible to Berkson's bias. The case mix and risk factor estimates observed in our study may not be directly generalizable to the wider Chinese population. Future studies employing a population-based design are warranted to validate our findings. It is important to note that the study population is from a region with a relatively low mean BMI. This leaner body-habitus distribution resulted in a limited number of individuals with high body mass index in the cohort, creating a “restricted range of exposure.” Methodologically, this most likely led to an attenuation of the effect size of the association between BMI and preterm birth. Therefore, our study might not have fully captured the stronger association that could exist in a population with a broader BMI spectrum. Future research involving multi-center populations with diverse BMI distributions is needed to validate our findings and quantify this association more accurately. We acknowledge that our study may be subject to residual confounding, and its single-center design may affect generalizability. The use of self-reported BMI could lead to non-differential misclassification, likely biasing results toward the null, while the lack of direct visceral adiposity measures is a recognized constraint. Potential misclassification of metabolic status, also likely non-differential, was also noted. Despite these limitations, we have taken care to contextualize our findings and emphasize that they provide valuable hypothesis-generating insights, underscoring the need for future multi-center studies with more precise measurements to confirm our results. Beside, the diagnosis of metabolic abnormalities in early pregnancy carries a risk of over-diagnosis. Physiological adaptations of early pregnancy, such as hemodilution from plasma volume expansion, vasodilation induced by progesterone, and stimulation of thyroid function by human chorionic gonadotropin (hCG), can alter metabolic parameters away from their pre-pregnancy baselines. These adaptations mean that the most diagnostic criteria we employed (such as blood pressure, glucose), which are based on thresholds derived from non-pregnant populations or fixed cut-offs, may have reduced specificity in the context of pregnancy, erroneously categorizing some normal physiological adaptations as pathological states. Future research aimed at establishing pregnancy-specific and gestational-age-adjusted diagnostic criteria for metabolic parameters would greatly enhance the accuracy of such studies.

In conclusion, our study shows that obesity and metabolic disorders have multiplicative and additive interaction effect on PTB and are linked to an increased risk of PTB. Aside from that, metabolic disorders may make normal-weight women more vulnerable to PTB. Therefore, clinical monitoring and treatment of the pregnant woman's metabolism, together with appropriate risk classification and improved prevention, are necessary in addition to focus on overweight or obesity.

## Data Availability

The raw data supporting the conclusions of this article will be made available by the authors, without undue reservation.
