# Supplementary material for: Maternal obesity phenotype, metabolic dysfunction, and preterm birth: a prospective birth cohort study
Source: Front Nutr. 2025 Oct 22;12:1648996. doi: 10.3389/fnut.2025.1648996 (PMC12585940; doi:10.3389/fnut.2025.1648996)
Supplement: Supplementary file 1 [file Table_1.docx]

**Maternal obesity phenotype, metabolic dysfunction, and preterm birth: A prospective birth cohort study**

**Supplementary Material**

**Fig S1. Flowchart of study population**

**Supplementary Table 1. Baseline Characteristics of Included and Excluded Study Participants**

**Supplementary Table 2. The Odds Ratios (ORs) for spontaneous PTB according to the body mass index, metabolic components, and metabolic status**

**Supplementary Table 3 Relationship of metabolic phenotypes and spontaneous PTB in different body weight phenotypes**

**Supplementary Table 4. Demographic, metabolic and clinical variables in pregnant women by obesity metabolic phenotype group (BMI categorization was based on WHO international cut-off values)**

**Supplementary Table 5. The Odds Ratios (ORs) for PTB according to the body mass index, metabolic components, and metabolic status (BMI categorization was based on WHO international cut-off values)**

**Supplementary Table 6. Relationship of metabolic phenotypes and PTB in different body weight phenotypes (BMI categorization was based on WHO international cut-off values)**

**Supplementary Table 7. Interaction analysis of the effects of overweight (including obese) and metabolically unhealthy on PTB (BMI categorization was based on WHO international cut-off values)**


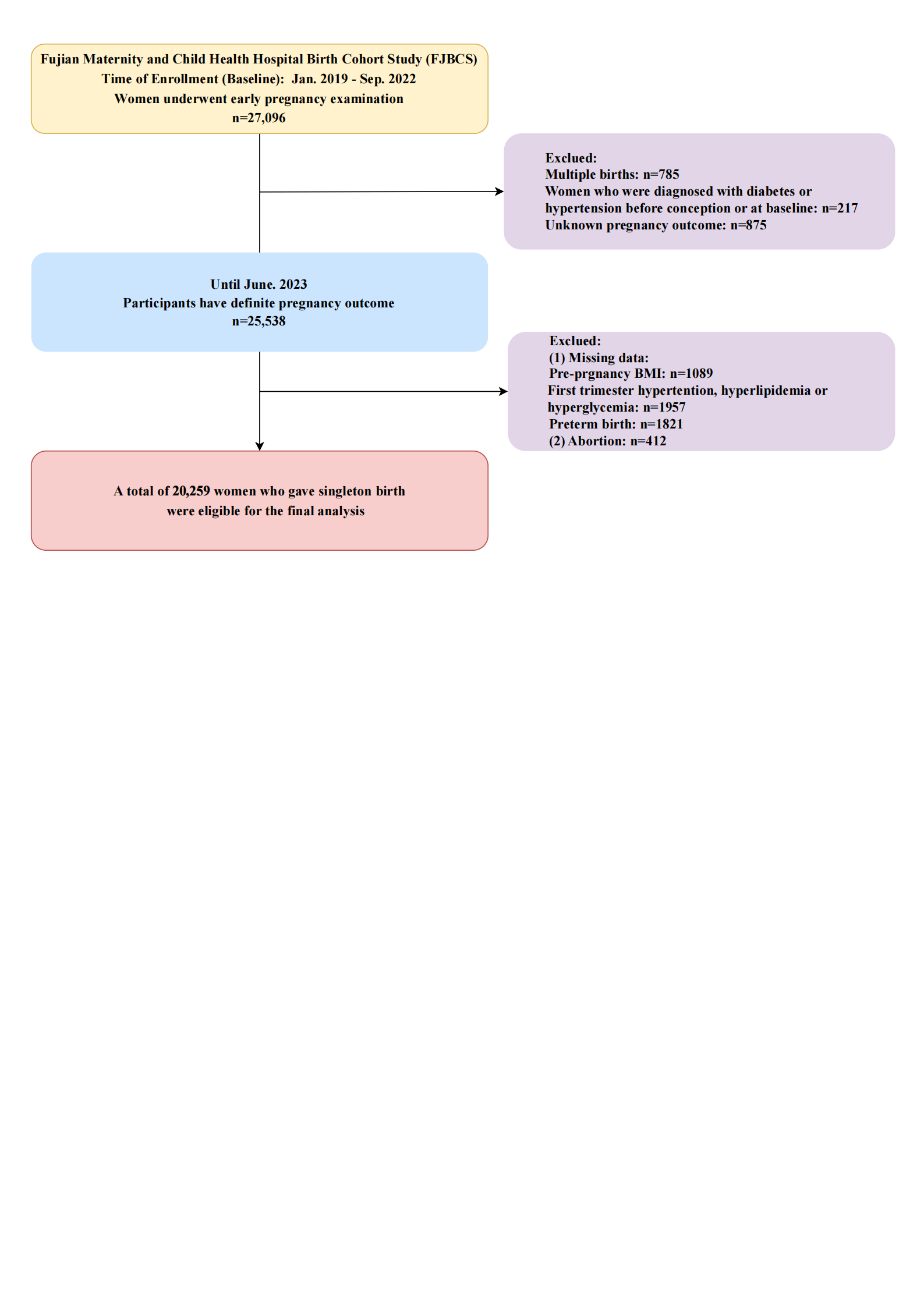


**Fig S1. Flowchart of study population**

**Supplementary Table 1. Baseline Characteristics of Included and Excluded Study Participants**

|  | **Total**  **(27096)** | **Non-participants**  **(6837)** | **Participants**  **(20259)** | ***P* value** |
| --- | --- | --- | --- | --- |
| Age | 30.3 ± 4.1 | 30.2 ± 4.4 | 30.3 ± 4.0 | 0.071 |
| Ethnicity-Han, n (%) | 26480 (97.9) | 6657 (97.6) | 19823 (98) | 0.639 |
| Educational-University level, n (%) | 18959 (70.1) | 4422 (64.8) | 14537 (71.9) | < 0.001 |
| Marriage, n (%) | 25488 (94.3) | 6257 (91.8) | 19231 (95.2) | < 0.001 |
| Smoking, n (%) | 608 (2.2) | 198 (2.9) | 410 (2) | < 0.001 |
| Alcohol Status, n (%) | 21331 (78.7) | 5096 (74.5) | 16235 (80.1) | < 0.001 |
| Assisted reproduction, n (%) | 2240 (8.4) | 816 (12.2) | 1424 (7.1) | < 0.001 |
| Gravidity, n (%) |  |  |  | < 0.001 |
| 1 | 11573 (43.4) | 2704 (42.4) | 8869 (43.8) |  |
| 2 | 7956 (29.9) | 1813 (28.4) | 6143 (30.3) |  |
| ≥3 | 7108 (26.7) | 1862 (29.2) | 5246 (25.9) |  |
| Parity, n (%) |  |  |  | < 0.001 |
| 0 | 16082 (60.4) | 3959 (62.1) | 12123 (59.8) |  |
| 1 | 9392 (35.3) | 2099 (32.9) | 7293 (36) |  |
| ≥2 | 1163 (4.4) | 321 (5) | 842 (4.2) |  |

Continuous variables with normal distributions are expressed as the mean-standard deviation.

**Supplementary Table 2. The Odds Ratios (ORs) for spontaneous PTB according to the body mass index, metabolic components, and metabolic status**

| **Variable** | **Total, n** | **PTB, n (%)** | **Crude** |  | **Adjusted** |  |
| --- | --- | --- | --- | --- | --- | --- |
|  |  |  | **OR (95%CI)** | ***P* value** | **OR (95%CI)** | ***P* value** |
| **Body Mass Index** |  |  |  |  |  |  |
| Underweight | 3114 | 109 (3.5) | 0.98 (0.79~1.21) | 0.858 | 1.06 (0.86~1.32) | 0.574 |
| Normal weight | 14112 | 502 (3.6) | 1(Ref) |  | 1(Ref) |  |
| Overweight（including obese） | 3033 | 107 (3.5) | 1.01 (0.82~1.25) | 0.926 | 0.97 (0.78~1.2) | 0.747 |
| **Metabolic Components** |  |  |  |  |  |  |
| Hypertensive |  |  |  |  |  |  |
| No | 18202 | 643 (3.5) | 1(Ref) |  | 1(Ref) |  |
| Yes | 2057 | 75 (3.6) | 1.05 (0.82~1.33) | 0.719 | 1.04 (0.81~1.33) | 0.746 |
| Hyperlipidemia |  |  |  |  |  |  |
| No | 15028 | 476 (3.2) | 1(Ref) |  | 1(Ref) |  |
| Yes | 5231 | 242 (4.6) | 1.5 (1.28~1.76) | <0.001 | 1.44 (1.22~1.69) | <0.001 |
| Hyperglycemia, |  |  |  |  |  |  |
| No | 19574 | 682 (3.5) | 1(Ref) |  | 1(Ref) |  |
| Yes | 360 | 15 (4.2) | 1.21 (0.72~2.04) | 0.477 | 1.10 (0.65~1.86) | 0.719 |
| Number of metabolically unhealthy components |  |  |  |  |  |  |
| 0 | 13489 | 429 (3.2) | 1(Ref) |  | 1(Ref) |  |
| 1 | 5819 | 237 (4.1) | 1.3 (1.11~1.53) | 0.001 | 1.25 (1.06~1.47) | 0.007 |
| 2 | 794 | 39 (4.9) | 1.62 (1.16~2.27) | 0.005 | 1.55 (1.1~2.17) | 0.012 |
| 3 | 46 | 2 (4.3) | 1.46 (0.35~6.06) | 0.602 | 1.35 (0.32~5.63) | 0.678 |
| **Metabolic Status** |  |  |  |  |  |  |
| Metabolically healthy | 13489 | 429 (3.2) | 1(Ref) |  | 1(Ref) |  |
| Metabolically unhealthy | 6770 | 289 (4.3) | 1.37 (1.18~1.6) | <0.001 | 1.32 (1.13~1.54) | <0.001 |
| Models were adjusted for age, ethnicity, education, marriage, smoking, alcohol status, gravidity, parity, assisted reproduction, income, work and inter-pregnancy  Abbreviations: preterm birth, PTB; odds ratio, OR; adjusted odds ratio, aOR. | | | | | | |

**Supplementary Table 3. Relationship of metabolic phenotypes and spontaneous PTB in different body weight phenotypes**

| **Variable** | **Total, n** | **PTB, n (%)** | **Crude** |  | **Adjusted** | |
| --- | --- | --- | --- | --- | --- | --- |
|  |  |  | **OR (95%CI)** | ***P* value** | **aOR (95%CI)** | ***P* value** |
| Metabolically healthy underweight | 2446 | 110 (4.5) | 1(Ref) |  | 1(Ref) |  |
| Metabolically unhealthy underweight | 668 | 25 (3.7) | 0.83 (0.53~1.29) | 0.397 | 0.8 (0.51~1.26) | 0.34 |
| Metabolically healthy normal weight | 10108 | 437 (4.3) | 1(Ref) |  | 1(Ref) |  |
| Metabolically unhealthy normal weight | 4985 | 298 (6) | 1.41 (1.21~1.64) | <0.001 | 1.34 (1.15~1.57) | <0.001 |
| Metabolically healthy overweight (including obese) | 935 | 41 (4.4) | 1(Ref) |  | 1(Ref) |  |
| Metabolically unhealthy overweight (including obese) | 1117 | 89 (8) | 1.89 (1.29~2.76) | 0.001 | 1.8 (1.22~2.65) | 0.003 |

Models were adjusted for age, ethnicity, education, marriage, smoking, alcohol status, gravidity, parity, assisted reproduction, income, work and inter-pregnancy

Abbreviations: OR, odds ratio; aOR, adjusted odds ratio.

**Supplementary Table 4. Demographic, metabolic and clinical variables in pregnant women by obesity metabolic phenotype group (BMI categorization was based on WHO international cut-off values)**

| **Maternal characteristics** | **Total**  **(20259)** | **Obesity metabolic phenotype** | | | | ***P* value** | |
| --- | --- | --- | --- | --- | --- | --- | --- |
|  |  | **MHUW/MHNW**  **(12554)** | **MHO**  **(935)** | **MUUW/MUNW**  **(5653)** | **MUO**  **(1117)** |  |  |
| Maternal age, years | 30.3 ± 3.9 | 29.9 ± 3.8 | 31.0 ± 4.0 | 30.8 ± 4.0 | 31.2 ± 4.2 | | < 0.001 |
| Ethnicity-Han, n (%) | 19823 (97.8) | 12284 (97.8) | 918 (98.2) | 5531 (97.8) | 1090 (97.6) | | 0.519 |
| Educational-University level, n (%) | 14537 (71.9) | 9184 (73.2) | 629 (67.3) | 4018 (71.2) | 706 (63.3) | | < 0.001 |
| Marriage, n (%) | 19231 (94.9) | 11867 (94.5) | 906 (96.9) | 5380 (95.2) | 1078 (96.5) | | < 0.001 |
| Smoking, n (%) | 410 ( 2.0) | 243 (1.9) | 31 (3.3) | 102 (1.8) | 34 (3) | | 0.007 |
| Alcohol Status, n (%) | 16235 (80.1) | 10028 (79.9) | 746 (79.8) | 4568 (80.8) | 893 (79.9) | | < 0.001 |
| Assisted reproduction, n (%) | 18638 (92.0) | 11717 (93.3) | 861 (92.1) | 5070 (89.7) | 990 (88.6) | | < 0.001 |
| Gravidity, n (%) |  |  |  |  |  | | < 0.001 |
| 1 | 8870 (43.8) | 5850 (46.6) | 369 (39.5) | 2303 (40.7) | 348 (31.2) | |  |
| 2 | 6143 (30.3) | 3784 (30.1) | 275 (29.4) | 1693 (29.9) | 391 (35) | |  |
| ≥3 | 5246 (25.9) | 2920 (23.3) | 291 (31.1) | 1657 (29.3) | 378 (33.8) | |  |
| Parity, n (%) |  |  |  |  |  | | < 0.001 |
| 0 | 12123 (59.8) | 7859 (62.6) | 513 (54.9) | 3184 (56.3) | 567 (50.8) | |  |
| 1 | 7293 (36.0) | 4228 (33.7) | 371 (39.7) | 2212 (39.1) | 482 (43.2) | |  |
| ≥2 | 801 ( 4.0) | 443 (3.5) | 48 (5.1) | 248 (4.4) | 62 (5.6) | |  |
| Pre-pregnancy BMI, kg/m^2^ | 21.2 ± 4.1 | 20.3 ± 2.1 | 27.7 ± 9.8 | 21.0 ± 2.1 | 27.8 ± 7.7 | | < 0.001 |
| SBP, mmHg | 114.5 ± 11.1 | 111.9 ± 9.7 | 114.9 ± 10.3 | 118.8 ± 12.1 | 122.5 ± 10.7 | | < 0.001 |
| DBP, mmHg | 69.3 ± 9.9 | 67.6 ± 7.9 | 69.7 ± 7.9 | 72.3 ± 12.8 | 74.4 ± 9.3 | | < 0.001 |
| FPG, mmol/L | 5.0 ± 1.2 | 5.0 ± 1.1 | 5.1 ± 1.3 | 5.1 ± 1.2 | 5.2 ± 1.3 | | < 0.001 |
| TC, mmol/L | 6.6 ± 1.2 | 6.5 ± 1.1 | 6.0 ± 1.1 | 7.0 ± 1.4 | 6.4 ± 1.3 | | < 0.001 |
| TG, mmol/L | 3.2 (2.5, 4.1) | 3.0 (2.4, 3.8) | 3.2 (2.5, 3.8) | 3.6 (2.9, 4.7) | 3.8 (3.0, 4.9) | | < 0.001 |
| HDL, mmol/L | 1.8 ± 0.4 | 1.8 ± 0.4 | 1.8 ± 0.3 | 1.8 ± 0.4 | 1.7 ± 0.3 | | < 0.001 |
| LDL, mmol/L | 3.6 ± 1.0 | 3.5 ± 0.9 | 3.2 ± 0.8 | 3.8 ± 1.2 | 3.4 ± 1.0 | | < 0.001 |
| Apo A1, g/L | 1.8 ± 0.3 | 1.8 ± 0.3 | 1.7 ± 0.3 | 1.8 ± 0.3 | 1.8 ± 0.3 | | < 0.001 |
| Apo B, g/L | 1.2 ± 0.3 | 1.2 ± 0.3 | 1.1 ± 0.2 | 1.3 ± 0.3 | 1.2 ± 0.3 | | < 0.001 |
| Apo B/Apo A1 | 0.5 ± 0.1 | 0.5 ± 0.1 | 0.5 ± 0.1 | 0.5 ± 0.1 | 0.6 ± 0.2 | | < 0.001 |
| Hypertensive, n (%) | 360 ( 1.8) | 0 (0) | 0 (0) | 257 (4.6) | 103 (9.3) | | < 0.001 |
| Hyperglycemia, n (%) | 2057 (10.2) | 0 (0) | 0 (0) | 1670 (29.5) | 387 (34.6) | | < 0.001 |
| Hyperlipidemia, n (%) | 5231 (25.8) | 0 (0) | 0 (0) | 4336 (76.7) | 895 (80.1) | | < 0.001 |
| Metabolic unhealthy, n (%) | 6770 (33.4) | 0 (0) | 0 (0) | 5653 (100) | 1117 (100) | | 6770 (33.4) |
| Preterm birth, n (%) | 1000 ( 4.9) | 547 (4.4) | 41 (4.4) | 323 (5.7) | 89 (8) | | < 0.001 |

Abbreviations: BMI, body mass index; SBP, systolic blood pressure; DBP, diastolic blood pressure; FPG, fasting plasma glucose; TC, total cholesterol; TG, triglyceride; HDL, high-density lipoprotein; LDL, low-density lipoprotein; Apo A1, apolipoprotein A1; Apo B, apolipoprotein B; MHUW, metabolically healthy underweight; MUUW, metabolically unhealthy underweight; MHNW, metabolically healthy normal weight; MUNW, metabolically unhealthy normal weight; MHO, metabolically healthy overweight (including obese); MUO, metabolically unhealthy overweight (including obese).

Continuous variables with normal distributions are expressed as the mean-standard deviation, whereas those with non-normal distributions are presented as the median and interquartile range.

**Supplementary Table 5. The Odds Ratios (ORs) for PTB according to the body mass index, metabolic components, and metabolic status (BMI categorization was based on WHO international cut-off values)**

| **Variable** | **Total, n** | **PTB, n (%)** | **Crude** |  | **Adjusted** |  |
| --- | --- | --- | --- | --- | --- | --- |
|  |  |  | **OR (95%CI)** | ***P* value** | **OR (95%CI)** | ***P* value** |
| **Body Mass Index** |  |  |  |  |  |  |
| Underweight | 3114 | 135 (4.3) | 0.89 (0.73~1.07) | 0.203 | 0.96 (0.79~1.16) | 0.681 |
| Normal weight | 15093 | 735 (4.9) | 1(Ref) |  | 1(Ref) |  |
| Overweight（including obese） | 2052 | 130 (6.3) | 1.32 (1.09~1.6) | 0.005 | 1.25 (1.03~1.52) | 0.025 |
| **Metabolic Components** |  |  |  |  |  |  |
| Hypertensive |  |  |  |  |  |  |
| No | 18202 | 877 (4.8) | 1(Ref) |  | 1(Ref) |  |
| Yes | 2057 | 123 (6) | 1.26 (1.03~1.53) | 0.021 | 1.23 (1.01~1.5) | 0.036 |
| Hyperlipidemia |  |  |  |  |  |  |
| No | 15028 | 657 (4.4) | 1(Ref) |  | 1(Ref) |  |
| Yes | 5231 | 343 (6.6) | 1.53 (1.34~1.76) | <0.001 | 1.44 (1.25~1.65) | <0.001 |
| Hyperglycemia, |  |  |  |  |  |  |
| No | 19574 | 950 (4.9) | 1(Ref) |  | 1(Ref) |  |
| Yes | 360 | 22 (6.1) | 1.28 (0.82~1.97) | 0.273 | 1.14 (0.73~1.76) | 0.567 |
| Number of metabolically unhealthy components |  |  |  |  |  |  |
| 0 | 13489 | 588 (4.4) | 1(Ref) |  | 1(Ref) |  |
| 1 | 5819 | 324 (5.6) | 1.29 (1.13~1.49) | <0.001 | 1.23 (1.07~1.42) | 0.004 |
| 2 | 111 | 16 (14.4) | 3.7 (2.16~6.32) | <0.001 | 3.33 (1.93~5.73) | <0.001 |
| 3 | 794 | 67 (8.4) | 2.02 (1.55~2.63) | <0.001 | 1.85 (1.42~2.42) | <0.001 |
| **Metabolic Status** |  |  |  |  |  |  |
| Metabolically healthy | 13489 | 588 (4.4) | 1(Ref) |  | 1(Ref) |  |
| Metabolically unhealthy | 6770 | 412 (6.1) | 1.42 (1.25~1.62) | <0.001 | 1.34 (1.18~1.53) | <0.001 |
| Models were adjusted for age, ethnicity, education, marriage, smoking, alcohol status, gravidity, parity, assisted reproduction, income, work and inter-pregnancy.  Abbreviations: PTB, preterm birth; OR, odds ratio; aOR, adjusted odds ratio. | | | | | | |

**Supplementary Table 6. Relationship of metabolic phenotypes and PTB in different body weight phenotypes (BMI categorization was based on WHO international cut-off values)**

| **Variable** | **Total, n** | **PTB, n (%)** | **Crude** |  | **Adjusted** | |
| --- | --- | --- | --- | --- | --- | --- |
|  |  |  | **OR (95%CI)** | ***P* value** | **aOR (95%CI)** | ***P* value** |
| Metabolically healthy underweight | 2446 | 110 (4.5) | 1(Ref) |  | 1(Ref) |  |
| Metabolically unhealthy underweight | 668 | 25 (3.7) | 0.83 (0.53~1.29) | 0.397 | 0.8 (0.51~1.26) | 0.34 |
| Metabolically healthy normal weight | 10108 | 437 (4.3) | 1(Ref) |  | 1(Ref) |  |
| Metabolically unhealthy normal weight | 4985 | 298 (6) | 1.41 (1.21~1.64) | <0.001 | 1.34 (1.15~1.57) | <0.001 |
| Metabolically healthy overweight (including obese) | 935 | 41 (4.4) | 1(Ref) |  | 1(Ref) |  |
| Metabolically unhealthy overweight (including obese) | 1117 | 89 (8) | 1.89 (1.29~2.76) | 0.001 | 1.8 (1.22~2.65) | 0.003 |

Models were adjusted for age, ethnicity, education, marriage, smoking, alcohol status, gravidity, parity, assisted reproduction, income, work and inter-pregnancy.

Abbreviations: OR, odds ratio; aOR, adjusted odds ratio.

**Supplementary Table 7. Interaction analysis of the effects of overweight (including obese) and metabolically unhealthy on PTB (BMI categorization was based on WHO international cut-off values)**

| **Measures** | **OR/Estimates** | **95% CI** | ***P* value** |
| --- | --- | --- | --- |
| Obesity metabolic phenotype |  |  |  |
| MHNW | 1(Ref) |  |  |
| MUNW | 0.95 | 0.69 | 1.32 |
| MHO | 1.26 | 1.09~1.45 | 1.45 |
| MUO | 1.72 | 1.36~2.18 | <0.001 |
| Subgroup analysis |  |  |  |
| Metabolically unhealthy on PTB |  |  |  |
| Normal weight | 1.26 | 1.09~1.45 | <0.001 |
| Overweight | 1.81 | 1.24~2.65 | <0.001 |
| Overweight on PTB |  |  |  |
| Metabolically healthy | 0.95 | 0.69~1.32 | 0.76 |
| Metabolically unhealthy | 1.36 | 1.07~1.75 | 0.01 |
| Interaction analysis |  |  |  |
| Multiplicative interaction | 1.44 | 0.96 | 2.16 |
| Additive interaction |  |  |  |
| RERI | 0.51 | 0.01~1.01 | 0.02 |
| AP | 0.30 | 0.05~0.55 | 0.01 |
| SI | 3.42 | 1.02~20.52 | <0.001 |

Multiplicative interaction of the effects of overweight (including obese) and metabolically unhealthy on preterm birth was assessed by including the main effects of them and the product term in the model and the P_interaction_ was represented by the p-value of product term.

RERI, AP were used to indicate additive interaction of overweight (including obese) and metabolically unhealthy on preterm birth.

Models were adjusted for age, ethnicity, education, marriage, smoking, alcohol status, gravidity, parity, assisted reproduction, income, work and inter-pregnancy.

Abbreviations: metabolically healthy normal weight; MHNW, normal weight with metabolic abnormalities; MUNW, overweight (including obese) without metabolic abnormalities; MHO, overweight (including obese) with metabolic abnormalities; MUO overweight (including obese) without metabolic abnormalities; Models adjusted for age, ethnicity, education, marriage, smoking, alcohol status, gravidity, production, and assisted reproduction
